# Effects of Different Pre-drying and Drying Methods on Volatile Compounds in the Pericarp and Kernel of *Amomum tsao-ko*

**DOI:** 10.3389/fpls.2022.803776

**Published:** 2022-02-25

**Authors:** Hui-wei Qin, Tian-mei Yang, Shao-bing Yang, Mei-quan Yang, Yuan-zhong Wang, Jin-yu Zhang

**Affiliations:** ^1^Medicinal Plants Research Institute, Yunnan Academy of Agricultural Sciences, Kunming, China; ^2^College of Traditional Chinese Medicine, Yunnan University of Chinese Medicine, Kunming, China

**Keywords:** *Amomum tsao-ko*, pericarp, kernel, drying method, volatiles

## Abstract

The effects of twelve different pre-drying and drying methods on the chemical composition in the pericarp and kernel of *Amomum tsao-ko* were studied. The volatile components were isolated from the samples by simultaneous distillation and extraction and analyzed by gas chromatography-mass spectrometry (GC-MS). Sixty and thirty-eight compounds were identified from pericarp and kernel, respectively, and the main constituents were oxygenated monoterpenes. These compounds were not only significantly affected by pre-drying and drying methods but also varied in content due to different tissue locations. The total volatile content of pericarp varied from 0.70 to 1.55%, with the highest obtained by microwave-dried samples (150 W) and the lowest in freeze-dried samples. The total volatile content of the kernel varied from 6.11 to 10.69%, with the highest content obtained during sun drying (SD) and the lowest content in samples treated with boiling water for 2 min. Oxygenated monoterpenes were the highest compounds in pericarp and kernel, which were also the most affected by drying methods. The highest content of oxygenated monoterpenes in the pericarp (0.77%) could be obtained by boiling water treatment for 5 min, and the highest content of oxygenated monoterpenes in the kernel (7.48%) could be obtained by SD. Additionally, the main components such as 1,8-cineole, 2-carene, (*Z*)-citral, nerolidol, (*Z*)-2-decenal, (*E*)-2-dodecenal, citral, (*E*)-2-octenal, 4-propylbenzaldehyde, and phthalan showed remarkable variations in pre-drying and drying methods.

## Introduction

*Amomum tsao-ko* Crevost et Lemaire is a perennial herbaceous plant of the genus *Amomum* (Zingiberaceae), widely distributed in southwest China and other parts of Asia. The dried fruit of *A. tsao-ko*, commonly known as “Cao-Guo” in Chinese, is well-known for its edible and medicinal properties (Yang et al., 2010). *A. tsao-ko* is commonly used as spices in hotpot, meat cooking, and many kinds of soup to remove peculiar smell and improve flavor and organoleptic properties (Wu et al., 2012; He et al., 2020), and as traditional Chinese medicines for treating dyspepsia, spleen and stomach deficiency cold, cough phlegm, diarrhea, and malaria (Qin et al., 2021). Modern pharmacological studies on *A. tsao-ko* have revealed a broad range of bioactivities such as antidiabetic, antimicrobial, antioxidant, antiobesity, antiinflammatory, antiproliferative, and neuroprotective effects (Zhang et al., 2014; Cui et al., 2017; He et al., 2020). Diverse phytochemicals including terpenes, aliphatics, aromatics, phenols, and flavonoids have been reported from *A. tsao-ko* (Zhang et al., 2014; Wang et al., 2021).

In China, *A. tsao-ko* is mainly distributed in Yunnan and a small amount in Guangxi and Guizhou. The planting area has reached 1,198,800 hm^2^, and the annual output is approximately 1,400 t in Yunnan, accounting for more than 90% of Chinese planting area and output (Yang et al., 2017). *A. tsao-ko*, a kind of industrial crop, is playing an important role in poverty alleviation in Yunnan mountainous areas. With the increase of planting area and yield year by year, primary processing of *A. tsao-ko* becomes more and more important to facilitate storage and transportation and retain more nutrients and medicinal ingredients and improve the quality of fruit. Different processing methods have important effects on the chemical composition of *A. tsao-ko* (Wang et al., 2021). In the current actual production, the first processing of planting farmers on *A. tsao-ko* is mostly drying. Drying effectively preserves fruit and prolongs shelf life for *A. tsao-ko*. Drying prevents the growth of spoilage microorganisms, slows enzyme activity, and slows many moisture-mediated reactions (Garcia-Segovia et al., 2011). In contrast, it can cause some alterations in aroma and prevent nutrient loss, color changes, and the formation of oxidation products (Hossain et al., 2010; Ozdemir et al., 2017). These positive and negative effects during the drying process depend on drying conditions such as temperature, time, environment, and the use of special equipment. Volatile compounds are the most sensitive components in food and medicinal material drying (Calín-Sánchez et al., 2013). According to Díaz-Maroto et al. (2003), the effect of a particular drying method on the release or retention of volatile compounds is not predictable and depends on the compound and spice concerned. The effect of drying on the volatile components of aromatic and medicinal plants has been the subject of many studies. Calvo-Irabien et al. (2009) compared shade drying, sun drying (SD), and oven-drying at 20°C and 40°C and showed that drying treatments had no effect on qualitative or quantitative characteristics of the *Lippia graveolens* essential oil. Yousif et al. (2000) reported that the level of thymol in vacuum-microwave-dried plants was 1.3 times the hot air-dried and had better color to *L. berlandieri*. Shahhoseini et al. (2013) investigated the effect of drying air temperature (30°C, 40°C, and 50°C) and air-flow rate (0.5, 1, and 1.5 m/s) on the quantity and quality of the essential oil of *Lippia citriodora*. In that research, the maximum essential oil content and the majority of the compounds were obtained at 50°C and 0.5 m/s air flow.

At present, most farmers will simply dry or bake *A. tsao-ko* before selling, and their understanding of the dried product quality is not deep enough. In addition, there are few reports on the effects of different drying treatments on the components in the pericarp and kernel of *A. tsao-k*o. In this study, fresh *A. tsao-k*o samples collected from Yunnan were used as raw materials, and the fruits were conducted on boiling water treatment, microwave drying, oven-drying, freeze-drying, air-drying (AD), SD, smoke drying, and electric baking drying. The chemical components in the pericarp and kernel were extracted and analyzed by simultaneous distillation-extraction and gas chromatography-mass spectrometry. The content differences of chemical components in different treatments were studied in order to provide data basis for the processing, preservation, and other application of *A. tsao-k*o industry, as well as the theoretical basis for the unification of *A. tsao-k*o production and drying standards and the enrichment of *A. tsao-k*o product types in the later stage.

## Materials and Methods

### Plant Materials

Approximately 50 kg of fresh and mature fruits were collected from Nujiang planting land in Yunnan. All the samples were identified as the fruit of *A. tsao-ko* Crevost et Lemarie of Zingiberaceae by Dr. Jinyu Zhang (Medicinal Plants Research Institute, Yunnan Academy of Agricultural Sciences, Kunming, China). The moisture content of the fruit was immediately measured on arrival, which was determined using the oven method (105°C for 24 h) in triplicate. Approximately 100 g of fruit samples were randomly selected and roasted to a constant weight, and the initial moisture content of *A. tsao-ko* was 78.5% on a wet basis.

### Pre-drying and Drying Methods

The hundred-grain weight information of the fruit was measured on the day of harvest. In addition, the samples were randomly divided into 12 groups, and the weight of each group was also weighed. A total of five pre-drying methods were used to treat samples including boiling water treatment for 2 min (2BW), boiling water treatment for 5 min (5BW), boiling water treatment for 10 min (10BW), microwave heating at 500 W for 10 min (MH), and oven heating at 105°C for 30 min (OH). The pretreatment time was selected in conjunction with local growers and processing plants. The five pretreated samples were then dried in the sun under the same conditions. A total of five drying methods including AD, SD, oven-drying (OD) at 50°C, microwave drying at 150 W (MD), and freeze-drying at −50°C (FD) were used to dry the other five groups of fresh samples. The moisture content was determined by the weight of the sample before and after drying. Two drying methods, namely, electric baking drying at 50°C (EBD) and smoke drying (SMD), were performed on local growers due to the lack of drying facilities, and the samples were from the same source. The moisture content of the two drying methods was measured by the hundred-grain weight because the farmers used the mass drying method. The drying process lasted until the moisture content of *A. tsao-ko* reached below 6% (wet basis), and the required drying time was recorded. The 5 pre-drying methods were independent of the other 7 drying methods and did not implement the factorial design. Three biological replicates were performed for each treatment condition.

### Simultaneous Distillation-Extraction

Dichloromethane was used as the extraction solvent, and naphthalene was used as the internal standard substance. They were purchased from Tianjin Fuyu Fine Chemical Co., Ltd. (Tianjin, China). The pericarp and kernel were separated and ground to a fine powder using a beater. Powder samples were uniformly screened through 30 mesh screens. The following experimental parameters were selected by comparing the results of the compound chromatogram in cross pre-experiment. First, a 5.0-g powder sample was added to a 1,000 ml flask with 350 ml of pure water and 3 ml of naphthalene (0.1 mg/ml), and 30 ml of dichloromethane was added to a 100 ml extraction bottle to serve as the extraction solvent. Then, the mixed solution and dichloromethane were heated to their respective boiling points and maintained for 2.0 h after refluxing. Subsequently, the obtained compound dissolved in the dichloromethane was cooled to room temperature and concentrated to 1.0 ml on a rotary evaporator. Finally, the sample was stored at 4°C for further analysis.

### Gas Chromatography-Mass Spectrometry Analysis

Gas Chromatography-Mass Spectrometry was carried out on an Agilent Technologies 7890A-5975C GC-MS unit with a DB-5MS column (30.00 m × 0.25 mm × 0.25 μm). The injection volume was 1.0 μl, and the injector temperature was 230? Helium was used as carrier gas at a constant flow rate of 1 ml/min with a split ratio of 40:1. The oven temperature was kept at 40°C for 1 min initially, then increased to 130°C at a rate of 10°C/min, held for 5 min, and reached a final temperature of 230°C at a rate of 8°C/min and held for 3 min. The transfer line temperature was 290°C, the ion source temperature was 230°C, and the quadrupole temperature was 150°C, operating in electron ionization mode (70 eV). The scan range was 50–600 *m/z* with full scan mode. The compounds were identified by comparison of their mass spectra with the NIST 11.0 library (including Wiley and Mainlib) data bank and confirmed by comparison with those published in the literature (He et al., 2013; Cui et al., 2017; Hu et al., 2019; Wang et al., 2021). The amounts (mg/g) of the compounds were calculated by peak area and naphthalene content.

### Statistical Analysis

The heat map and network diagram of correlation were constructed based on the concentration of identified volatiles using the R-3.2 software. Metabolites were mapped onto biochemical pathways according to annotation in Kyoto Encyclopedia of Genes and Genomes (KEGG).

## Results and Discussion

### Effects of Pre-dying and Drying Method on Volatiles

As shown in Supplementary Figure 1, the color of dried fruit varies greatly. Overall, the fruit color presented by the four drying methods, namely, boiling water pretreatment, freeze-drying, electric baking, and smoke drying differs greatly from other drying methods, which may be related to drying methods, energy transfer methods, and water loss rate. Fruit dried in shade and sun had the slowest water loss because it was greatly affected by natural conditions and unstable heat transfer (Supplementary Table 1). Fumigation was also a slow process by which the heat of the smoke between the fruits removed moisture and gave the fruit a complex smoky smell. Microwave drying took the shortest time because the microwave is a kind of high-frequency electromagnetic wave with a short oscillation period and strong penetration ability. Microwave radiation, as an internal heating method, can accelerate the evaporation of water inside the fruit.

The volatile components of the dried fruits were extracted and analyzed in order to study the effect of the pre-drying and drying methods on the volatiles of *A. tsao-ko*. Results obtained were shown in Supplementary Tables 2, 3. Overall, 60 and 38 compounds were identified from pericarp and kernel, respectively, with 26 compounds in common. The main volatile components of pericarp were 1,8-cineole, (*Z*)-2-decenal, citral, (*E*)-2-dodecenal, (*Z*)-citral, β-pinene, and nerolidol. The main volatile components of the kernel were 1,8-cineole, (*Z*)-2-decenal, citral, (*E*)-2-dodecenal, (*Z*)-citral, 2-carene, geraniol, 4-propylbenzaldehyde, phthalan, nerolidol, α-phellandrene, citral, (*E*)-2-octenal, and α-methylcinnamaldehyde. He et al. (2013) identified 55–70 components of pericarp and 35–48 components of the kernel from three batches of *A. tsao-ko* volatile oil and found that 29 of them, such as 1,8-cineole, α-terpineol, α-pinene, β-pinene, and nerolidol, were common components of pericarp and kernel, which was similar with our results. Comparing our results with those of the author, it seems that some differences in the volatile composition could be attributed to the geographic origin of the plant and the methods of extraction. He et al. (2013) extracted volatile oil by hydrodistillation, whereas in this study, we used simultaneous distillation extraction to recover volatiles. The simultaneous distillation extraction process is conducted at lower temperatures, and the volatile components are extracted by dichloromethane when evaporating, so as to minimize the loss of volatile components and the decomposition of some thermal instability and oxidation components. Hydrodistillation is a developmental system that is prone to the loss of some low boiling point compounds and the destruction of thermal instability and oxidation components.

#### Effects on Volatiles in the Pericarp

**FIGURE 1:**
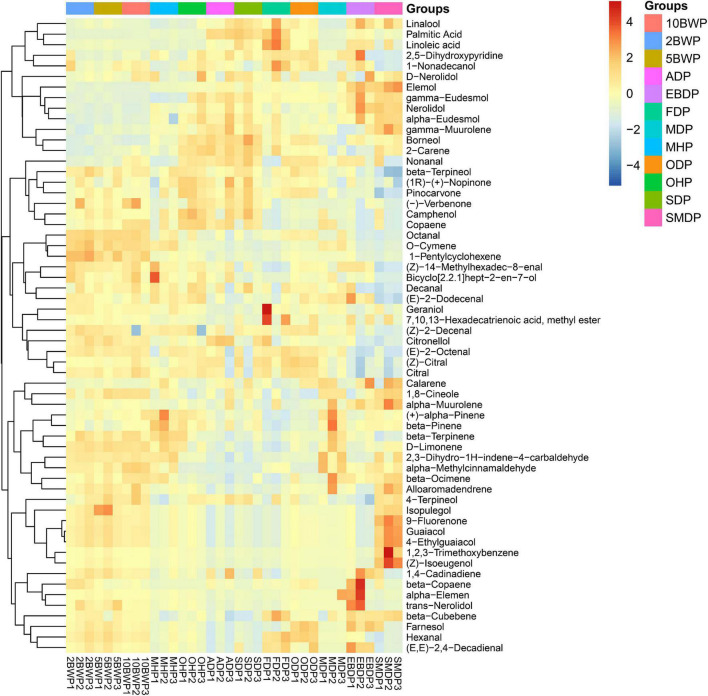
Heat map visualization of volatiles in the pericarp of *Amomum tsao-ko* with different pre-drying and drying methods.

Subsequently, the volatile compounds detected were visualized on the heat map to observe the overall changes in the chemical composition of *A. tsao-ko* under different pre-drying and drying methods. As shown in Figure 1 and Table 1, different from SD, the effect of pre-drying with boiling water, microwave (500 W), and oven (at 105°C) resulted in the increase of numerous monoterpene hydrocarbons, oxygenated monoterpenes, and aromatics in the pericarp, which was more pronounced by boiling water but less affected by treatment time. These compounds, such as *O*-cymene, 1-pentylcyclohexene, β-terpinene, *D*-limonene, β-ocimene, and α-methylcinnamaldehyde, were significantly increased in content after pre-drying. As we found that most of these compounds were also increased in the oven-drying (at 50°C), microwave drying (150 W), electric baking, and smoke drying but were significantly reduced in air-dried, sun-dried, and freeze-dried samples. These compounds were more abundant at high-temperature conditions, which may be related to complex chemical changes inside the fruit. Studies have suggested that microwave, oven at high temperature, and other high-temperature conditions can also cause low boiling point component volatilization, heat-sensitive component decomposition or some special reaction isomerization of epoxy compounds, oxidation of terpenoids, higher alkanes, organic acids and lipids, and other polymer compounds (Fan et al., 2021). Lemon verbena, for example, showed (*Z*)-β-ocimen when dried at 40°C and 60°C, but not when dried at 30°C (Shahhoseini et al., 2013). Tian et al. (2016) studied the changes of drying on volatile components of shiitake mushrooms and found that the samples of shiitake mushrooms with hot AD and microwave drying had 23 more components than the fresh samples, and the total amount of volatile compounds increased significantly, indicating that drying was, in fact, very significant in the generation of volatile compounds. Obviously, drying causes an increase in the amount of water moving in tissues, but reducing water movement also inhibits the diffusion of constituent molecules (Pirbalouti et al., 2013). Therefore, it is also possible that the strength of water evaporation in tissues of air-dried, sun-dried, and freeze-dried samples was too weak to increase the outflow of compound molecules, resulting in a decrease in the concentration of volatile components in the pericarp. In fact, high temperatures may accelerate compound metabolism, oxidation, or chemical rearrangement, leading to the increase or disappearance of certain volatiles and the emergence of novel molecules (Fan et al., 2021). Microwave (500 W and 150 W) could obtain higher (+)-α-pinene, β-pinene, β-terpinene, *D*-limonene, 2,3-dihydro-1H-indene-4-carbaldehyde, α-methylcinnamaldehyde, and β-ocimene. The reduction of minor constituents such as nonanal, 2-carene, borneol, γ-muurolene, α-eudesmol, nerolidol, γ-eudesmol, and elemol may be explained by the volatilization of compounds at excessive temperature during pre-drying with boiling water, microwave (500 W), and oven (at 105°C).

**TABLE 1:** Relative content of volatiles in pericarp of *Amomum tsao-ko*.

No.	Name	Relative content (%)											
		
		2BWP	5BWP	10BWP	MHP	OHP	ADP	SDP	FDP	ODP	MDP	EBDP	SMDP
1	Hexanal	–	–	–	–	–	–	–	0.003	0.005	–	0.001	–
2	(+)-α-Pinene	0.015	0.022	0.026	0.036	0.021	0.011	0.013	0.002	0.010	0.049	0.015	0.016
3	β-Terpinene	0.001	–	0.002	0.005	0.001	0.001	0.001	–	–	0.006	0.002	–
4	β-Pinene	0.030	0.033	0.044	0.074	0.042	0.027	0.028	0.003	0.024	0.101	0.036	0.033
5	Octanal	0.007	0.007	0.008	0.003	0.001	0.002	0.002	0.002	0.005	0.003	–	–
6	*O*-Cymene	0.008	0.006	0.007	0.006	–	0.001	–	–	–	0.007	–	–
7	*D*-Limonene	0.006	0.007	0.008	0.008	0.003	0.002	0.002	–	0.002	0.012	0.003	0.005
8	1,8-Cineole	0.166	0.229	0.218	0.128	0.070	0.094	0.099	0.045	0.075	0.223	0.094	0.148
9	β-Ocimene	–	–	0.004	0.004	–	0.001	0.001	–	0.001	0.008	0.004	0.006
10	(*E*)-2-Octenal	0.011	0.011	0.012	0.007	0.009	0.007	0.008	0.008	0.014	0.012	–	0.002
11	β-Terpineol	0.004	0.003	0.004	0.005	0.005	0.005	0.006	0.003	0.007	0.005	0.004	0.001
12	Guaiacol	–	–	–	–	–	–	–	–	–	–	–	0.007
13	1-Pentylcyclohexene	0.009	0.005	0.005	–	–	–	–	–	–	–	–	–
14	Isopulegol	–	0.006	–	–	–	–	–	–	–	–	–	0.006
15	Camphenol	–	–	–	0.001	0.006	0.004	0.005	–	–	0.001	–	0.002
16	(1*R*)-(+)-Nopinone	0.003	0.002	0.003	0.003	0.007	0.007	0.006	0.003	0.006	0.003	0.005	0.003
17	(*E*,*E*)-2,4-Decadienal	–	–	–	–	–	–	0.001	0.004	0.003	–	0.008	–
18	Pinocarvone	–	–	–	0.001	0.005	0.005	0.005	0.002	0.005	0.002	0.002	–
19	Linalool	–	–	–	–	0.001	0.006	0.008	0.005	0.006	–	0.010	0.008
20	Borneol	–	–	–	0.006	0.009	0.011	0.011	0.005	0.011	0.003	0.008	0.008
21	Non-anal	–	–	0.009	–	0.010	0.012	0.011	0.003	0.014	0.012	0.009	0.005
22	4-Terpineol	0.006	0.009	0.010	0.006	0.007	0.008	0.008	0.005	0.008	0.009	0.006	0.009
23	2-Carene	0.051	0.072	0.071	0.060	0.069	0.066	0.066	0.039	0.078	0.073	0.045	0.059
24	Decanal	0.002	0.003	0.005	0.007	0.006	0.004	0.006	0.003	0.005	0.006	0.003	–
25	(−)-Verbenone	0.003	–	0.006	–	0.003	0.005	0.005	0.001	–	–	0.001	0.001
26	Citronellol	–	–	–	–	–	0.006	0.002	0.002	–	–	–	–
27	(*Z*)-Citral	0.072	0.087	0.089	0.057	0.064	0.054	0.060	0.050	0.114	0.075	0.029	0.030
28	Geraniol	0.021	0.015	0.019	0.014	0.011	0.001	0.005	0.027	0.012	0.018	0.003	0.008
29	(*Z*)-2-Decenal	0.200	0.223	0.130	0.163	0.105	0.102	0.118	0.085	0.205	0.178	0.117	0.055
30	Citral	0.162	0.192	0.199	0.134	0.127	0.092	0.099	0.088	0.198	0.150	0.062	0.049
31	4-Ethylguaiacol	–	–	–	–	–	–	–	–	–	–	–	0.008
32	2,3-Dihydro-1H-indene-4-carbaldehyde	0.031	0.035	0.033	0.034	0.013	0.006	0.007	0.007	0.014	0.047	0.010	0.025
33	α-Methylcinnamaldehyde	0.019	0.022	0.035	0.017	–	–	–	0.007	–	0.035	0.015	0.017
34	1,2,3-Trimethoxybenzene	–	–	–	–	–	–	–	–	–	–	–	0.017
35	(*Z*)-Isoeugenol	–	–	–	–	–	–	–	–	–	–	–	0.016
36	Alloaromadendrene	–	–	0.002	–	–	–	–	–	–	0.005	–	0.007
37	(*E*)-2-Dodecenal	0.160	0.158	0.150	0.150	0.128	0.078	0.077	0.061	0.152	0.199	0.165	0.041
38	α-Muurolene	0.010	0.014	0.014	0.011	0.010	0.011	0.009	0.006	0.011	0.022	0.018	0.020
39	β-Cubebene	0.007	0.006	0.008	0.002	0.005	0.004	0.002	0.009	0.007	0.009	0.013	0.012
40	β-Copaene	0.026	0.005	0.007	–	–	0.009	0.003	0.004	0.019	–	0.042	0.006
41	γ-Muurolene	0.006	0.035	0.029	0.041	0.041	0.061	0.049	0.025	0.048	0.038	0.028	0.079
42	Elemol	0.003	0.003	–	0.011	0.012	0.017	0.015	0.010	0.020	0.017	0.045	0.045
43	Nerolidol	0.051	0.065	0.048	0.083	0.083	0.085	0.075	0.037	0.082	0.095	0.150	0.116
44	Calarene	–	–	–	0.007	–	0.006	0.004	0.002	0.007	0.012	0.011	0.010
45	α-Elemen	–	–	–	–	–	–	–	–	–	0.010	0.016	–
46	1,4-Cadinadiene	–	–	–	–	–	0.004	0.002	–	–	–	0.005	0.002
47	γ-Eudesmol	–	–	–	–	0.007	0.010	0.009	0.005	0.008	0.010	0.013	0.012
48	Copaene	–	–	0.006	0.002	0.006	0.006	0.004	–	–	0.005	–	0.002
49	2,5-Dihydroxypyridine	0.005	–	0.004	0.007	0.007	0.010	0.008	0.008	0.016	0.012	0.013	–
50	α-Eudesmol	0.017	0.024	0.022	0.023	0.035	0.034	0.031	0.020	0.031	0.041	0.040	0.040
51	(*Z*)-14-Methylhexadec-8-enal	0.015	0.012	0.014	0.016	0.009	0.008	0.005	–	0.012	0.012	0.014	–
52	9-Fluorenone	–	–	–	–	–	–	–	–	–	–	–	0.008
53	Farnesol	–	–	–	–	–	–	–	–	0.006	–	0.007	0.002
54	Bicyclo[2.2.1]hept-2-en-7-ol	0.022	0.014	0.023	0.030	0.013	0.012	0.005	0.003	0.011	0.014	0.010	–
55	Palmitic Acid	–	–	0.005	0.012	0.008	0.074	0.074	0.067	0.046	0.006	0.035	0.016
56	*D*-Nerolidol	–	–	0.004	0.004	0.003	0.004	0.006	0.002	0.002	0.004	0.005	0.006
57	trans-Nerolidol	0.002	0.003	–	–	–	–	–	–	–	–	0.011	–
58	1-Non-adecanol	0.005	0.002	0.006	–	0.006	0.006	0.003	0.008	0.009	0.002	0.009	–
59	Linoleic acid	–	–	–	–	–	0.006	0.011	0.017	0.010	–	0.015	0.004
60	7,10,13-Hexadecatrienoic acid, methyl ester	–	–	–	–	–	–	0.002	0.011	0.003	–	0.007	0.003

*Data are shown as mean (n = 3).*

The samples with oven-drying (at 50°C) showed similar changes in expression of pericarp components to pre-drying with oven (at 105°C), but the former contained important increases in the concentration of farnesol, hexanal, linalool, and palmitic acid, and the decrease of these compounds might be caused by the higher oven temperature. Meanwhile, the palmitic acid and linoleic acid contents of oven-dried (at 50°C) samples were higher than pre-drying with oven (at 105°C), and lower than air-dried, sun-dried, and freeze-dried samples, which confirmed that low temperature helped to protect fatty acid compounds. The volatile compounds originated mostly from chemical or enzymatic oxidation of unsaturated fatty acids and further interactions with proteins, peptides, and free amino acids. The heat may have accelerated the chemical conversion of fatty acid compounds. Similarly, the same changes of chemical composition in pericarp were observed in samples with electric baking and smoke drying, and the two drying methods also resulted in an important reduction in oxygenated monoterpenes including citral, (*Z*)-citral, citronellol, (*Z*)-2-decenal, β-terpineol, and camphenol. Nevertheless, 1,4-cadinadiene, β-copaene, α-elemen, and trans-nerolidol were reached their highest content by electric baking drying. Alloaromadendrene, 4-terpineol, and isopulegol reached the maximum amount in smoke drying, and 9-fluorenone, 1,2,3-trimethoxybenzene, and (*Z*)-isoeugenol appeared only in smoked samples. Smoke drying significantly increases the role of internal organization enzymes in products, which achieves the aim of slow drying and hot working by exposing fruits in the smoke produced by burning wood, leaves, and waste products from crops. This drying method may enable fruits to absorb smoke composition to produce some additional ingredients or generate synthetic products through the material, and smoke components interact in long-time smoked processing (Yao et al., 2005). Hu et al. (2019) also found that the compounds in fumigated *A. tsao-ko* were richer and more complex, but the corresponding compound content was lower, which was consistent with our experimental results.

#### Effects on Volatiles in Kernel

**FIGURE 2:**
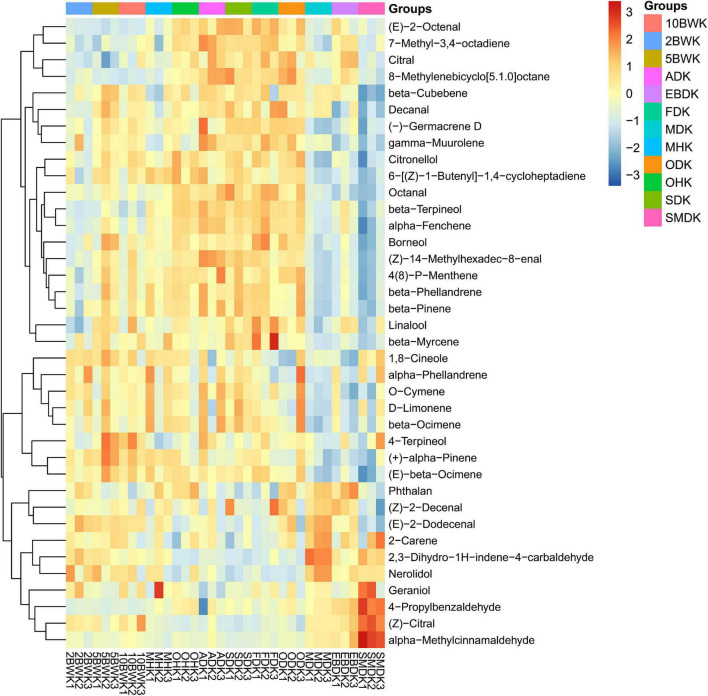
Heat map visualization of volatiles in the kernel of *A. tsao-ko* with different pre-drying and drying methods.

Figure 2 and Table 2 show the results of the cluster heat map based on 38 chemical components detected from the kernel of *A. tsao-ko*. Different from the pericarp, most of the chemical components in the kernel were decreased by boiling water treatment at 2 min but increased after 5 min and 10 min. Among them, monoterpenes as borneol, (+)-α-pinene, and (*E*)-β-ocimene reached the highest content in boiling water treatment for 5 min. However, β-terpineol, 4-propylbenzaldehyde, citral, and some aliphatics as (*E*)-2-octenal, 7-methyl-3,4-octadiene, and 8-methylenebicyclo[5.1.0]octane were decreased after boiling water treatment. These compounds may have a stronger affinity with water, or their boiling point may be declined by miscibility with water, thus they were lost with water during drying (Sellami et al., 2011). Pre-drying with microwave (500 W) also resulted in a reduction of these compounds, as well as β-cubebene, γ-muurolene, and 4-terpineol. However, Wang et al. (2021) found that high-temperature drying increased the monoterpene hydrocarbon content of *A. tsao-ko*, such as α-thujene, α-pinene, sabinene, cymene, and terpinolene. Comparing our results with those of these authors, it seems that some differences in variation of composition could be attributed to the drying methods and the methods of extraction. Pre-drying with oven (at 105°C) and SD was similarly expressed in compounds from the kernel, where the lower (*E*)-2-dodecenal, 2,3-dihydro-1H-indene-4-carbaldehyde, nerolidol, and (*Z*)-citral were observed. In addition, SD also caused a decrease in phthalan, (*Z*)-2-decenal, and 2-carene. Oven-drying is to absorb heat from the surface of the material and then transfer it to the interior by means of convection circulation of hot air (Abraham and Sparrow, 2004). This external heating method had a greater impact on the composition in the pericarp than the kernel in a short time of pre-drying. As compared with oven heating, boiling water infiltrates the fruit internal change tissue cell osmotic pressure to promote the flow of molecular. Microwave, an internal heating method, also makes the internal molecular severe collision and friction material in a very short period of oscillation of microwave high-frequency electric field (Mashkani et al., 2018). Therefore, these two drying methods have more significant changes in the composition of the kernel in a short time.

**TABLE 2:** Relative content of volatiles in kernel of *A. tsao-ko*.

No.	Name	Relative content (%)											
		2BWK	5BWK	10BWK	MHK	OHK	ADK	SDK	FDK	ODK	MDK	EBDK	SMDK
1	(+)-α-Pinene	0.022	0.030	0.022	0.025	0.007	0.007	0.011	0.005	0.007	0.009	–	0.011
2	β-Phellandrene	0.003	0.005	0.002	0.007	–	0.004	0.008	0.003	0.004	–	–	–
3	(*E*)-β-Ocimene	0.011	0.011	0.008	0.007	–	–	–	0.003	0.003	–	–	–
4	β-Pinene	0.005	0.006	0.003	0.008	–	0.011	0.010	0.003	0.005	–	–	0.003
5	Octanal	0.029	0.032	0.027	0.035	0.051	0.044	0.059	0.048	0.043	0.031	0.023	0.032
6	α-Phellandrene	0.094	0.072	0.073	0.123	0.067	0.092	0.122	0.037	0.089	0.027	0.055	0.118
7	*O*-Cymene	0.030	0.019	0.020	0.031	0.020	0.032	0.035	0.017	0.024	0.015	0.013	0.026
8	*D*-Limonene	0.046	0.045	0.040	0.061	0.039	0.051	0.076	0.035	0.043	0.018	0.030	0.057
9	1,8-Cineole	1.367	1.758	1.571	2.052	2.166	1.659	2.180	1.538	1.520	1.237	1.120	1.950
10	β-Ocimene	0.025	0.025	0.019	0.040	0.031	0.038	0.048	0.027	0.031	0.004	0.014	0.028
11	(*E*)-2-Octenal	0.050	0.046	0.049	0.088	0.165	0.148	0.199	0.156	0.139	0.068	0.107	0.091
12	β-Terpineol	0.018	0.019	0.011	0.033	0.048	0.045	0.050	0.039	0.042	0.024	0.033	0.026
13	Linalool	–	0.013	0.007	–	–	–	0.011	0.017	0.010	0.003	0.015	0.014
14	α-Fenchene	0.014	0.016	0.010	0.017	0.025	0.029	0.026	0.024	0.025	0.017	0.021	0.014
15	Borneol	0.005	0.009	–	–	–	–	–	0.008	0.008	0.002	–	–
16	7-Methyl-3,4-octadiene	0.019	0.013	0.019	0.020	0.040	0.047	0.047	0.038	0.033	0.021	0.040	0.037
17	4-Terpineol	0.037	0.049	0.051	0.037	0.042	0.048	0.048	0.040	0.046	0.044	0.045	0.066
18	2-Carene	0.257	0.317	0.304	0.362	0.410	0.352	0.416	0.312	0.366	0.341	0.337	0.401
19	Decanal	0.014	0.027	0.017	0.009	0.017	0.021	0.028	0.023	0.024	0.027	0.009	0.012
20	Citronellol	0.007	0.008	0.010	0.003	0.011	–	0.008	0.005	0.014	–	0.005	0.004
21	4(8)-P-Menthene	0.002	0.007	0.002	0.003	0.003	0.008	0.005	–	0.014	–	–	–
22	(*Z*)-Citral	0.484	0.526	0.655	0.585	0.612	0.531	0.601	0.510	0.516	0.551	0.669	0.975
23	Geraniol	0.153	0.131	0.176	0.234	0.218	0.105	0.207	0.183	0.165	0.169	0.156	0.271
24	(*Z*)-2-Decenal	0.947	1.170	1.214	1.460	1.693	1.315	1.848	1.386	1.460	1.220	1.384	1.174
25	Citral	1.038	1.167	1.456	1.763	2.362	2.027	2.499	1.942	2.062	1.425	1.821	1.530
26	4-Propylbenzaldehyde	0.147	0.161	0.165	0.257	0.366	0.177	0.329	0.212	0.274	0.242	0.335	0.507
27	8-Methylenebicyclo[5.1.0]octane	0.037	0.017	0.015	0.041	0.090	0.139	0.158	0.096	0.136	0.036	0.091	0.037
28	2,3-Dihydro-1H-indene-4-carbaldehyde	0.093	0.091	0.081	0.099	0.049	0.063	0.054	0.057	0.054	0.152	0.096	0.153
29	Phthalan	0.213	0.205	0.200	0.307	0.394	0.241	0.284	0.202	0.311	0.266	0.308	0.240
30	β-Myrcene	0.008	0.015	0.012	0.015	–	–	0.030	0.042	0.004	–	–	–
31	α-Methylcinnamaldehyde	0.058	0.063	0.080	0.062	0.085	0.075	0.057	0.056	0.074	0.106	0.134	0.293
32	6-[(*Z*)-1-Butenyl]-1,4-cycloheptadiene	0.007	0.017	0.010	0.029	0.025	0.010	0.024	–	0.018	–	–	–
33	(*E*)-2-Dodecenal	0.584	0.697	0.701	0.784	0.791	0.702	0.841	0.624	0.752	0.731	0.683	0.560
34	(−)-Germacrene D	0.008	0.012	0.011	0.005	–	0.011	0.022	0.013	0.019	–	0.005	0.005
35	β-Cubebene	0.021	0.029	0.023	0.011	0.021	0.028	0.030	0.015	0.027	0.029	0.028	0.011
36	γ-Muurolene	0.024	0.026	0.027	0.010	0.017	0.048	0.044	0.037	0.037	0.026	0.007	0.012
37	Nerolidol	0.235	0.266	0.250	0.293	0.286	0.241	0.257	0.207	0.214	0.307	0.274	0.321
38	(*Z*)-14-Methylhexadec-8-enal	0.003	0.008	0.004	–	0.003	0.023	0.018	0.004	0.007	–	–	–

*Data are shown as mean (n = 3).*

The composition changes in the kernel of air-dried, freeze-dried, and oven-dried (at 50°C) samples were roughly the same as sun-dried samples, and most of the compounds in the samples had high content. But oven-drying reduced the contents of 1,8-cineole, α-phellandrene, *O*-cymene, *D*-limonene, β-ocimene, 2,3-dihydro-1H-indene-4-carbaldehyde, and nerolidol. Interestingly, although phthalan, (*Z*)-2-decenal, (*E*)-2-dodecenal, 2-carene, 2,3-dihydro-1H-indene-4-carbaldehyde, nerolidol, geraniol, and (*Z*)-citral were decreased in air-dried and freeze-dried samples, most of these compounds increased significantly in the microwave drying (150 W), electric baking, and smoke drying. Among them, microwave drying (150 W) obtained more phthalan, (*E*)-2-dodecenal, 2-carene, 2,3-dihydro-1H-indene-4-carbaldehyde, and nerolidol. Geraniol, 4-propylbenzaldehyde, (*Z*)-citral, and α-methylcinnamaldehyde reached the highest content in smoke drying. However, most of the compounds upregulated in air-dried, freeze-dried, and oven-dried (at 50°C), and sun-dried samples were downregulated in the microwave drying (150 W), electric baking, and smoke drying. Aldehydes, the most abundant chemical family in different dry-cured meats, are probably the most interesting lipid-derived volatile compounds due to their ability to produce a wide range of flavors and odors (Lorenzo and Carballo, 2015). In this study, high amounts of aromatic compounds were found in smoke drying samples. These compounds were accumulated sufficiently to affect the smell of dried *A. tsao-ko*. Deng et al. (2015) also reported that lots of aromatics helped to build the odor of heat pump dried squids.

### Effects of Pre-dying and Drying Method on Main Volatiles

#### Effects on Main Volatiles in the Pericarp

**FIGURE 3:**
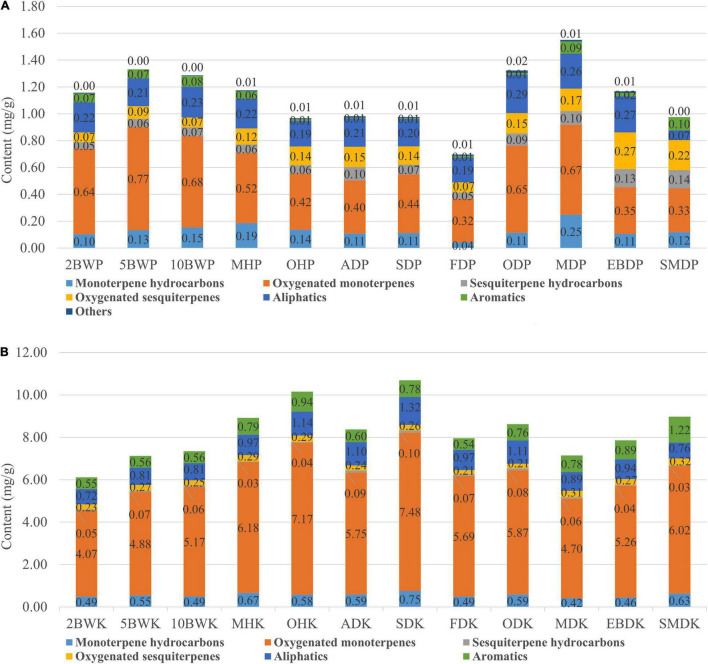
Changes in the proportion of the different chemical families of volatiles in the pericarp **(A)** and kernel **(B)** of *A. tsao-ko* with different pre-drying and drying methods.

Sixty compounds identified from pericarp contained 6 monoterpene hydrocarbons, 16 oxygenated monoterpenes, 9 sesquiterpene hydrocarbons, 7 oxygenated sesquiterpenes, 12 aliphatics, 9 aromatics, and 1 other compound. The concentration of these compounds was dramatically affected by pre-drying and drying methods. As shown in Figure 3A, the total contents of compounds in the pericarp reached the lowest content by freezing drying. Conversely, the highest increase in the total content was found in microwave drying (150 W), followed by pre-drying with boiling water (5 min). The lower temperature conditions of freeze-drying may lead to a decrease of enzymatic activity and downregulation or damage of metabolic pathways, causing the insufficient supply of precursor substances and a significant decline in compounds (Wang et al., 2021).

Quantitatively, the results obtained manifested that oxygenated monoterpenes represented the major chemical class in the pericarp. Our results showed that pre-drying with boiling water, oven (at 50°C), and microwave (150 and 500 W) obtained more oxygenated monoterpene contents in the pericarp, including the main component as 1,8-cineole, (*Z*)-citral, and citral, while pre-drying with oven (at 105°C), air, sun, freezing, electric baking, and smoke decreased the amounts of these compounds. This suggests that high temperature facilitates the synthesis of oxygenated monoterpenes, which was also demonstrated in drying experiments on Lemon verbena by Shahhoseini et al. (2013). In addition, the content of 1,8-cineole was significantly decreased using the oven (at 50°C and 105°C), indicating that oven-drying was not conducive to the synthesis and accumulation of this component in the pericarp. In addition to oxygenated monoterpenes, microwave treatment (150 W and 500 W) also increased the concentration of monoterpene hydrocarbons in the pericarp. The maximum content of the main component as β-pinene was reached by microwave at 150 W; however, the increase of microwave power also led to the decrease of monoterpene content. The pre-drying with boiling water also increased the content of monoterpene hydrocarbons, and the content increased with the prolonging of boiling water treatment time. Certain researchers have suggested that there may be membranes selectively more permeable to certain volatiles or separate compartments for the synthesis of emitted volatiles and stored substances (Gershenzon et al., 2000). The osmotic pressure difference caused by boiling water and the high-frequency motion caused by microwaves may have more significant effects on these membranes and compartments. Sesquiterpene hydrocarbons and oxygenated sesquiterpenes were compounds with larger molecular weights, higher boiling points, and lower volatility than monoterpenes. In this study, it was found that sesquiterpene hydrocarbons and oxygenated sesquiterpenes in pericarp were significantly reduced under the pretreatment at high temperatures, and their highest contents were reached by electric baking and smoke drying. Nerolidol, a major oxygenated sesquiterpene component, reached its maximum amount by electric baking drying. According to Díaz-Maroto et al. (2003), sometimes, substances of relatively low volatility, such as sesquiterpenes, are released more readily than more volatile compounds. It is worth noting that smoke drying increased aromatic compounds but significantly reduced aliphatic compounds in the pericarp, and these compounds seemed more likely to react or lose with smoke.

Compared with detected compounds, it was found that different pre-drying and drying methods had the most significant effects on the contents of *D*-limonene, 2-carene, 1,8-cineole, borneol, (*Z*)-citral, citral, nerolidol, and γ-eudesmol in pericarp, but had no significant effects on calarene and *D*-nerolidol.

#### Effects on Main Volatiles in Kernel

Thirty-eight compounds identified from the kernel contained 11 monoterpene hydrocarbons, 11 oxygenated monoterpenes, 3 sesquiterpene hydrocarbons, 1 oxygenated sesquiterpene, 6 aliphatics, and 6 aromatics. On the whole, the content of compounds in the kernel was higher than pericarp, such as the difference of main components including 1,8-cineole (22.0-fold), (*Z*)-2-decenal (15.7-fold), citral (25.3-fold), (*E*)-2-dodecenal (10.9-fold), (*Z*)-citral (10.1-fold), and nerolidol (3.4-fold) between kernel and pericarp in sun-dried samples. The results of the study by He et al. (2013) and Hu et al. (2019) also showed that the kernel of *A. tsao-ko* generally contains a higher level of compounds. In contrast, our results illustrated that the concentration of most compounds in the kernel varied dramatically with the pre-drying and drying methods, and this variation was different from the pericarp. As shown in Figure 3B, the total contents of compounds in the kernel reached the highest amount by SD, followed by pre-drying with oven (at 105°C). The lowest content was obtained by pre-drying with boiling water (2 min), followed by pre-drying with boiling water (5 min) and microwave (150 W). Combined with the total volatile content found that there seems to be a competitive relationship in compounds between pericarp and kernel. Studies have reported that plant reproductive resources are limited, and there is a trade-off between two or more organs competing for the same resource library (Campbell, 2000). More metabolizable energy accumulated in the pericarp may lead to reduced metabolite expression content in the kernel.

As far as the kernel is concerned, oxygenated monoterpenes were also the main chemical class. SD and pre-drying with oven at 105°C significantly increased oxygenated monoterpene contents in the kernel, including major components as 1,8-cineole, geraniol, (*Z*)-2-decenal, and citral. However, (*Z*)-citral, the main component, was decreased significantly by SD, pre-drying with oven at 105°C, and reached the highest content by smoke drying. Moreover, the highest monoterpene hydrocarbons and aliphatic contents in the kernel were obtained using the two methods. SD also yielded more sesquiterpene hydrocarbons. Most of the kernel compounds reached their maximum amounts in the sun-dried samples, including the main components such as 2-carene, (*E*)-2-octenal, and (*E*)-2-dodecenal. With oven-drying was different, the other four pre-drying methods including boiling water (2 min, 5min, and 10 min) and microwave (500 W) treatment significantly reduced the content of monoterpene hydrocarbons, oxygenated monoterpenes, aliphatics, and aromatics in the kernel, and this change was more obvious in the samples treated with boiling water, which may be related to drying medium and energy transfer. It is possible that boiling water hydrolyzes some synthases in the kernel, thus reducing the synthesis and accumulation of compounds, or water molecules affect the osmotic pressure of cells or even cell lysis, promoting the release of metabolites from kernel tissue or transfer to pericarp (Hanschen et al., 2018). Oven-drying, a method of external heating by hot air, arrived less heat energy to the kernel during a short period of pre-drying and, therefore, has less effect on kernel compounds. AD, freeze-drying, oven-drying, microwave drying (150 W), electric baking, and smoke drying brought about a certain extent decline in the contents of monoterpene hydrocarbons, oxygenated monoterpenes, sesquiterpene hydrocarbons, and aliphatics in the kernel compared with SD. Among them, microwave drying had the greatest effect on monoterpene hydrocarbons and oxygenated monoterpenes, while smoke drying had a more significant influence on sesquiterpene hydrocarbons and aliphatics. As with microwave drying (150 W), pre-drying with microwave (500 W) also led to the reduction of kernel compounds. Microwave radiation may damage the kernel tissue structure and destroy the metabolic function of the kernel. Another possibility was the compounds that were lost with water and transferred to the pericarp during the drying process. Internal heating caused by microwaves penetrating the interior of a wet material produced a higher water vapor pressure leading to an increased drying rate, which enhanced the migration of water from the material interior to its surface and the outward diffusion of other molecules (Calín-Sánchez et al., 2013). The lower kernel compounds in microwave drying (150 W) were associated with a longer treatment time. Lin et al. (1998) confirmed that heat damage suffered of a product is proportional to temperature and time involved during drying. Drying at a high temperature and long drying time usually leads to thermal injury and adversely affects texture, color, flavor, and constituent of products (Yongsawatdigul and Gunasekaran, 1996). Our results show that natural SD could obtain the highest content of kernel compounds, while most of the compounds were lost to varying degrees after artificial drying. Despite the loss of most of the components, smoke drying increased the content of aromatic compounds in the kernel, which was consistent with the effect of aromatic compounds in the pericarp. In addition, oxygenated sesquiterpenes, as nerolidol, in kernel reached its maximum content by smoke drying. Smoke drying is drying fruits by the smoke generated by the burning of wood or crop wastes. In this way, the fruits may absorb the aromatic components in the smoke during the long period of fumigation, so that the content of aromatic compounds was higher.

According to the ANOVA, the compound content including α-fenchene, β-terpineol, (*Z*)-citral, citral, octanal, (*E*)-2-octenal, 4-propylbenzaldehyde, and phthalan were most significant differences in the kernel with different pre-dying and drying methods, whereas β-phellandrene, β-pinene, α-phellandrene, decanal, and *O*-cymene had no obvious changes.

### Correlation Between Volatiles and Drying Methods

**FIGURE 4:**
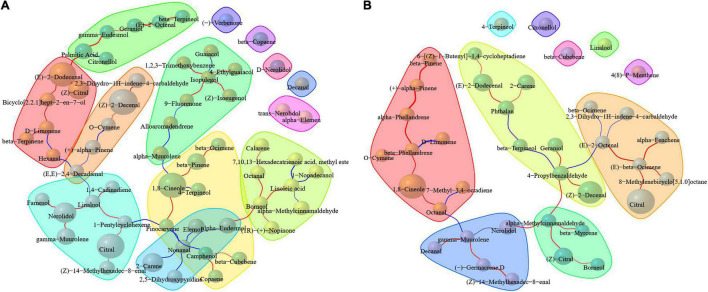
Correlation of volatiles in the pericarp **(A)** and kernel **(B)** of *A. tsao-ko* with different pre-drying and drying methods.

Correlation analysis of compounds was conducted based on Spearman correlation coefficient through the content of compounds in different groups in order to find the components that may have important effects on the metabolism or accumulation of compounds under different treatment methods. As shown in Figure 4A, palmitic acid, (*E*)-2-dodecenal, *D*-limonene, (*E*,*E*)-2,4-decadienal, linalool, nerolidol, 1-pentylcyclohexene, pinocarvone, nonanal, camphenol, borneol, linoleic acid, 1,8-cineole, and isopulegol in pericarp were correlated with various compounds. As far as the kernel is concerned, β-phellandrene, octanal, γ-muurolene, α-methylcinnamaldehyde, 4-propylbenzaldehyde, (*E*)-2-octenal, (*E*)-β-ocimene, and phthalan were observed to have more correlations with compounds (Figure 4B). Mapping these metabolites to KEGG revealed that *D*-limonene, linoleic acid, and (*E*)-2-octenal were all involved in metabolic pathways (map01100). *D*-Limonene, borneol, linoleic acid, 1,8-cineole, and (*E*)-2-octenal were all involved in the biosynthesis of secondary metabolites (map01110). *D*-Limonene, borneol, and 1,8-cineole were involved in monoterpenoid biosynthesis (map00902). *D*-Limonene was involved in limonene and pinene degradation (map00903) and biosynthesis of terpenoids and steroids (map01062). Linoleic acid was involved in linoleic acid metabolism (map00591) and biosynthesis of unsaturated fatty acids (map01040). When metabolites with different contents are involved in metabolic pathways, the final substances may lead to differences in the chemical composition of fruits, but the conversion mechanism of metabolites is extremely complex in plants. The biological mechanism and pathways of these metabolites under different pre-drying and drying conditions in *A. tsao-ko* are still poorly understood and should be further studied to better clarify the effects of drying techniques on the generation of volatile compounds.

**FIGURE 5:**
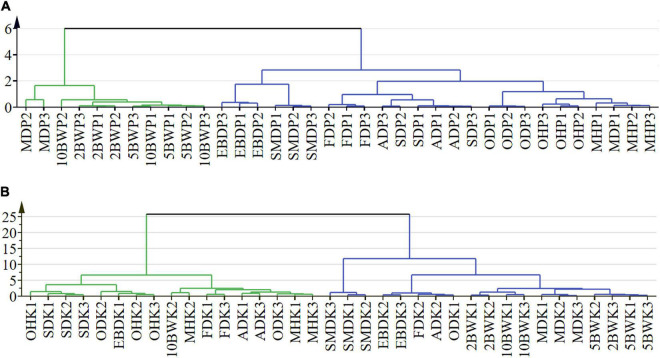
Cluster analysis of pre-drying and drying methods in the pericarp **(A)** and kernel **(B)** of *A. tsao-ko*.

Cluster analysis was carried out to determine the relationship between the different drying methods on the basis of their volatile compound contents (Figures 5A,B). Results showed that the existence of one well-defined group by boiling water treatment (2, 5, and 10 min) was related to the same treatment method. The closest clustering distance between microwave drying (150 W) and boiling water treatment was attributed to the similar component accumulation, especially in the main component of the kernel, which was also reflected in the heat map. The samples of electric baking and smoke drying had a close expression on pericarp and kernel compounds so that the two drying methods were close to each other. The similar effects of freeze-drying, AD, and SD for pericarp composition may be related to the relatively low drying temperature. For kernel, SD was close to pre-drying with oven (at 105°C). Freeze-drying and AD were close to pre-drying with microwave (500 W), while microwave (500 W) has a similar composition to oven (at 105°C) and oven (at 50°C) in the pericarp. Due to the relationship between energy transfer and biological characteristics of tissue, the effects of pre-drying and drying methods on pericarp and kernel composition were different, and this difference was more reflected in kernel composition.

## Conclusion

To sum up, different pre-drying and drying methods had different effects on the volatiles of pericarp and kernel. Among them, pericarp could obtain the highest volatile components under microwave drying at 150 W (MD), and kernel could obtain the highest volatile components under SD conditions. In some cases, pre-drying and drying by these methods increased the concentration of certain compounds. This was particularly the case of SD, which brought about remarkable increases in the content of most of the major volatile components, resulting in the highest total volatile content in the pericarp and kernel, but the drying time of this method was long, and the drying conditions were difficult to control. Oven-drying would seem to be more advisable, and in that, they were simple, fast, and low cost and helped to acquire the volatiles of fruits. In general, the volatile contents in the pericarp and kernel were higher under oven-drying condition. Obviously, oven-drying at 50°C (OD) was the best way to dry *A. tsao-ko* without pre-drying. When drying equipment is insufficient or drying time is not required in the actual processing process, the method of combining oven-drying and SD can be combined (OH), so as to save cost and obtain high volatile content. This study provided a data basis for analyzing the effects of different pre-drying and drying methods used by growers and processing plants on the volatiles in the pericarp and kernel of *A. tsao-ko* and a reference for relevant personnel to select specific drying methods to obtain preselected natural products.

## Data Availability Statement

The original contributions presented in the study are included in the article/Supplementary Material, further inquiries can be directed to the corresponding author/s.

## Author Contributions

H-WQ contributed to data curation and writing – review and editing. T-MY helped in conceptualization and project administration. S-BY contributed to the investigation and formal analysis. M-QY contributed to formal analysis. Y-ZW helped in review – editing. J-YZ contributed to supervision, project administration, and funding acquisition. All authors contributed to the article and approved the submitted version.

## Conflict of Interest

The authors declare that the research was conducted in the absence of any commercial or financial relationships that could be construed as a potential conflict of interest.

## Publisher’s Note

All claims expressed in this article are solely those of the authors and do not necessarily represent those of their affiliated organizations, or those of the publisher, the editors and the reviewers. Any product that may be evaluated in this article, or claim that may be made by its manufacturer, is not guaranteed or endorsed by the publisher.
