# Supplementary material for: Effects of Different Pre-drying and Drying Methods on Volatile Compounds in the Pericarp and Kernel of Amomum tsao-ko
Source: Front Plant Sci. 2022 Feb 25;13:803776. doi: 10.3389/fpls.2022.803776 (PMC8914167; doi:10.3389/fpls.2022.803776)
Supplement: Supplementary file 1 [file Data_Sheet_1.docx]

**
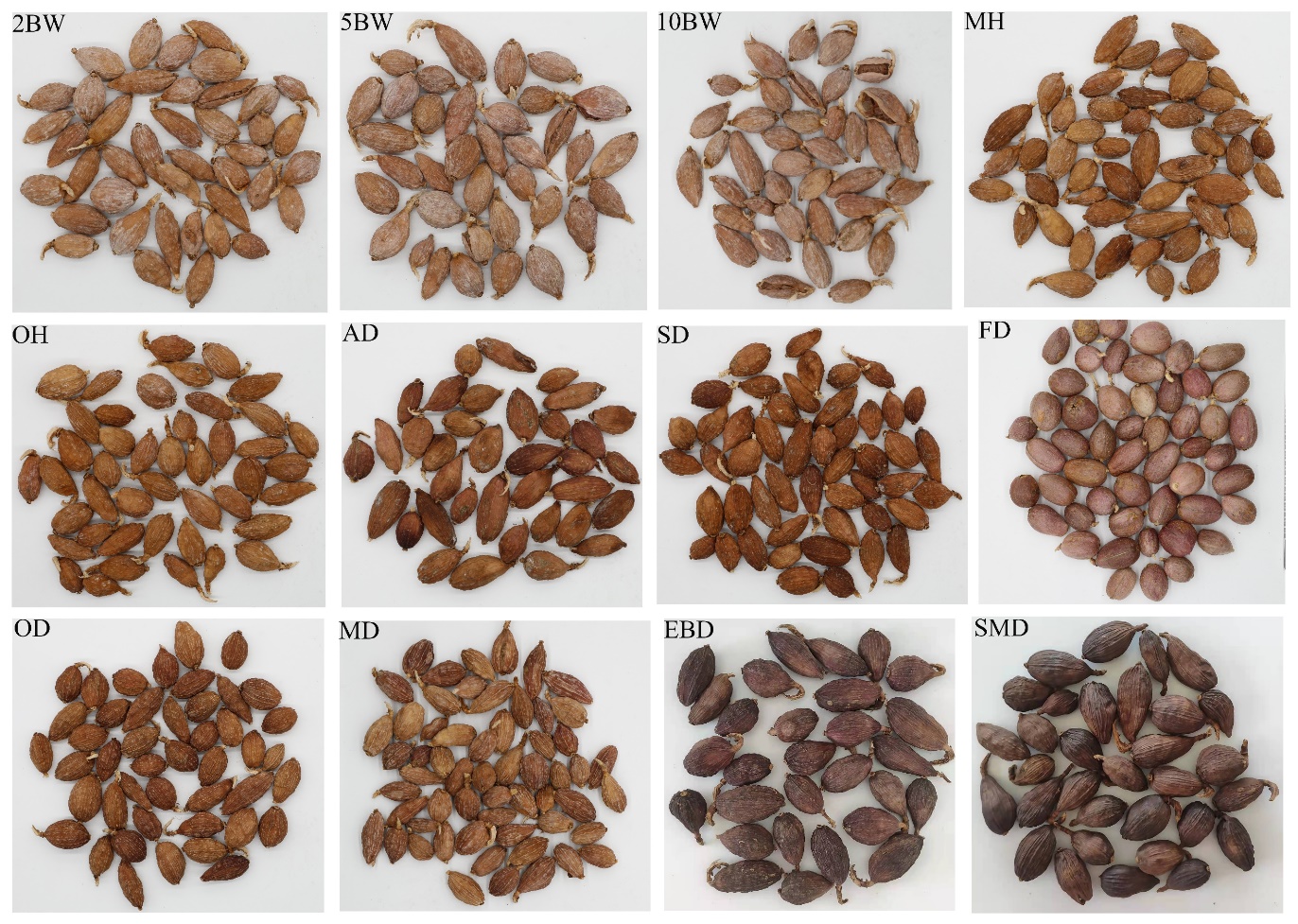
**

**FIGURE S1** *A. tsao-ko* with different pre-drying and drying methods.

**TABLE S1** The processing time of per-drying and drying methods.

| Drying method | Water loss rate (%) | Processing time |
| --- | --- | --- |
| 2BW | 76.31 | 18d |
| 5BW | 75.90 | 18d |
| 10BW | 76.53 | 18d |
| MH | 76.54 | 18d |
| OH | 75.89 | 18d |
| AD | 75.64 | 41d |
| SD | 76.18 | 18d |
| FD | 76.49 | 143h |
| OD | 76.42 | 53h |
| MD | 75.89 | 13h |
| EBD | 74.81 | 48h |
| SMD | 73.79 | 10d |
